# Microdrilled tapers to enhance optical fiber lasers for sensing

**DOI:** 10.1038/s41598-021-00046-7

**Published:** 2021-10-14

**Authors:** R. A. Perez-Herrera, M. Bravo, P. Roldan-Varona, D. Leandro, L. Rodriguez-Cobo, J. M. Lopez-Higuera, M. Lopez-Amo

**Affiliations:** 1 Department of Electrical Electronic and Communication Engineering, Public University of Navarra, 31006 Pamplona, Spain; 2 Institute of Smart Cities (ISC), Public University of Navarra, 31006 Pamplona, Spain; 3 Photonics Engineering Group, University of Cantabria, 39005 Santander, Spain; 4 CIBER-Bbn, Instituto de Salud Carlos III, 28029 Madrid, Spain; 5 Instituto de Investigacion Sanitaria Valdecilla (IDIVAL), 39005 Cantabria, Spain

**Keywords:** Fibre lasers, Imaging and sensing

## Abstract

In this work, an experimental analysis of the performance of different types of quasi-randomly distributed reflectors inscribed into a single-mode fiber as a sensing mirror is presented. These artificially-controlled backscattering fiber reflectors are used in short linear cavity fiber lasers. In particular, laser emission and sensor application features are analyzed when employing optical tapered fibers, micro-drilled optical fibers and 50 μm-waist or 100 μm-waist micro-drilled tapered fibers (MDTF). Single-wavelength laser with an output power level of about 8.2 dBm and an optical signal-to-noise ratio of 45 dB were measured when employing a 50 μm-waist micro-drilled tapered optical fiber. The achieved temperature sensitivities were similar to those of FBGs; however, the strain sensitivity improved more than one order of magnitude in comparison with FBG sensors, attaining slope sensitivities as good as 18.1 pm/με when using a 50 μm-waist MDTF as distributed reflector.

## Introduction

Today, fiber Bragg grating (FBG) based sensors are well-stablished transducers for a great number of sensing applications^1^. They are especially well suited for strain and temperature measurements. Thus, they are extensively used for monitoring civil works or in sensing platforms including a large number of this type of sensors^2^. Their main advantage is that the measured parameter is coded in a wavelength shift, which is immune to optical-power fluctuations. Moreover, because they are wavelength-selective reflectors, they can be employed as a part of an optical laser cavity. Due to this, since 1993 FBG sensing systems based on optical fiber lasers have been developed^3^. These lasing sensors have demonstrated a high sensitivity and a high optical signal-to-noise ratio (OSNR)^4^. Typically, FBG sensing systems offer a temperature sensitivity of 11 pm/$^\circ$C and a strain sensitivity of 1.2 pm/µε^5,6^. These sensitivities have been enough to establish this type of sensors as a flagship in sensing along more than two decades.

On the other hand, micro-drilling techniques, such as the employed in this work, have shown a high potential recently, not only in FBGs writing^7^, but also to develop enhanced optical fiber lasers and more sensitive sensing structures. Femtosecond (fs) laser can offer strong refractive index variations without the requirement of a photosensitivity enhancement technique^8^. This is achieved by using ultrashort pulses and extreme high instantaneous power. The fs laser can also induce non-linear multi-photon absorption of materials to locally change the refractive index of the core of a SMF. Thus, backscattering reflectors can be fabricated inside the core of an optical fiber. A number of laser generation applications of these artificially controlled backscattering fiber reflectors (ACBFRs) have been previously demonstrated. This type of distributed reflectors can be inscribed along different types of optical fiber. Previous works experimentally demonstrated their properties for distributed short-linear-cavity fiber lasers^9^ or random fiber laser generation^10^ assisted by ACBFRs inscribed along a single-mode fiber. On the other hand, it has also been experimentally demonstrated that multi-wavelength emission with single-longitudinal mode (SLM) behavior can be obtained when these distributed reflectors are inscribed not in a standard SMF but along a highly-doped erbium fiber^11^. It this case, ACBFRs act not only as distributed reflectors but also as saturable absorbers, obtaining up to eight simultaneous SLM emission lines.

In this work, we have developed quasi-randomly distributed reflectors inscribed into a standard single-mode or tapered optical fiber with different waist dimensions as a sensing mirror. Their performances in fiber laser configurations are experimentally evaluated. By using this type of mirrors, it is also possible to develop short-cavity fiber optic laser sensors which offer higher strain sensitivity than regular FBG transducers. In particular, the laser generation and sensor application features when optical tapered fibers (TF), micro-drilled optical fibers (MDF) or two different micro-drilled tapered fibers (MDTF) are presented.

## Working principle: inscription process

**Figure 1:**
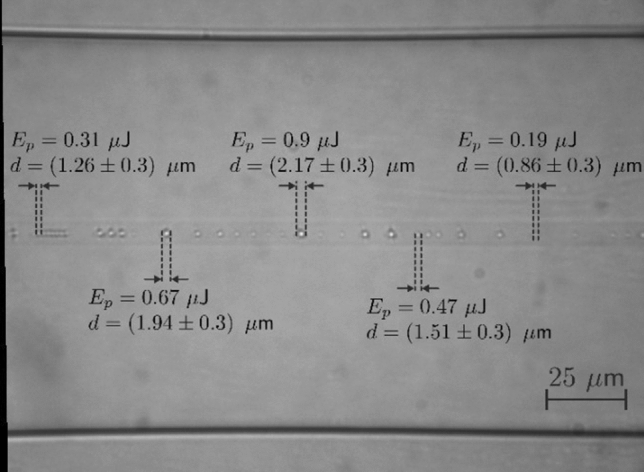
Microscope image of the quasi-randomly distributed reflector along the SMF.

In this experimental study, three different distributed reflectors based on tapered optical fibers or/and micro-drilled optical fibers were employed. A standard transversal inscription setup, like the one employed in^9^, was selected. The distributed reflectors have been manufactured using a femtosecond commercial Fiber Laser Chirped Pulse Amplifier (FLCPA) from CALMAR lasers, operating at 1030 nm wavelength, with 370 fs of pulse duration, and a variable pulse repetition (PRR) rate available up to 120 kHz. Femtosecond laser processing provides inhomogeneity enhancement of the refractive index of the fiber^12^, increasing the distributed scattering. The fiber, located over a nano-resolution Aerotech stage motor, is placed on a slide and covered with a coverslip. Between them, an index-matching oil is deposited to limit fiber-induced aberrations^13^. Then, the laser pulses are tightly focused through a 100$\times$/NA = 0.5 objective lens from Mitutoyo Corporation. Regarding the inscription parameters, it is worth noting that pseudo-randomly varying pulse energies between 0.19 and 0.9 µJ have been used (as can be seen in Fig. 1). Likewise, the period ($\Lambda$) between each laser spot is pseudo-randomly modified between 1 and 10 µm.

### Micro-drilled standard single-mode optical fiber (MDF)

In the first study, a micro-drilled standard single-mode optical fiber (MDF), with a 125 μm diameter was used. Figure 1 depicts a microscope image of the quasi-randomly distributed reflector along the fiber, showing the dimensions of some modifications induced along the SMF. As it may be seen in this figure, the random location of the induced changes was achieved by randomly modulating the pulse repetition rate at every pulse (which corresponds to 100 ms approximately). Consequently, a quasi-random inscribed structure with several micro-drilled points was attained. However, the repetition rate of the femtosecond laser could not be simultaneously modulated at the same rate as the pulse-inscription speed. Thus, direct random inscription was not possible, but quasi-randomly spaced spots was.

### Micro-drilled tapered optical fiber (MDTF): 100 µm-waist tapered fiber

**Figure 2:**
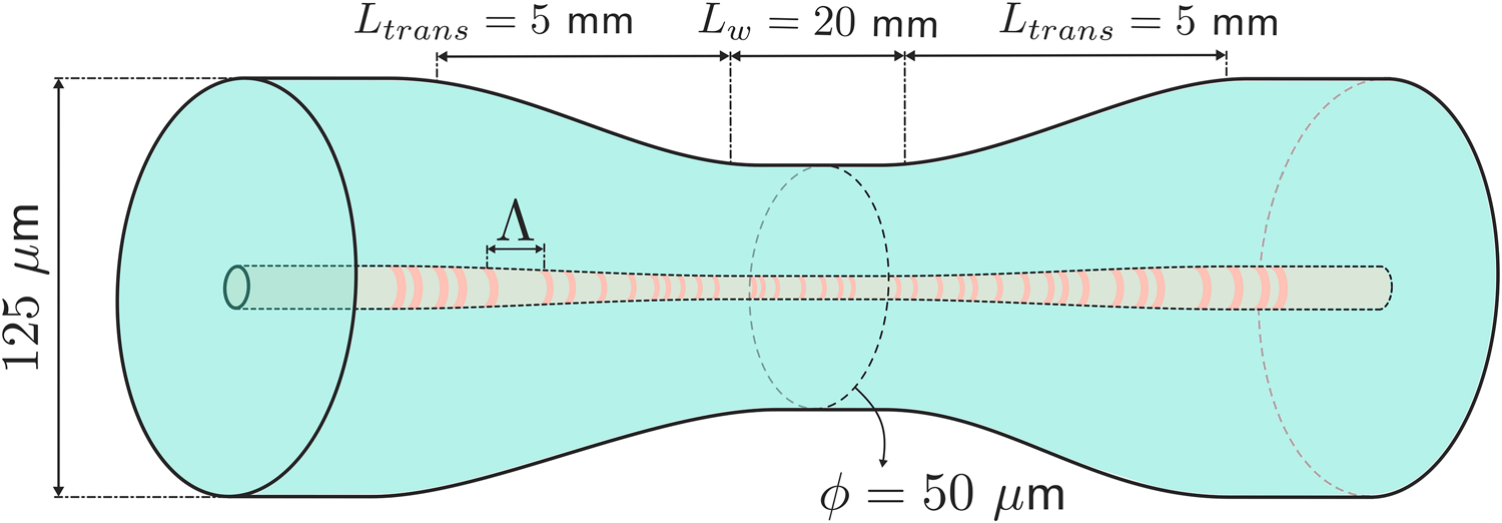
Schematic illustration of the 50 µm-waist tapered SMF, in which micro-modifications were applied using the femtosecond laser inscription setup.

Secondly, two different micro-drilled tapered optical fibers (MDTF) were developed. Both of them were fabricated from standard single-mode fiber (SMF). The fabrication of the tapered fibers was carried out by means of a Taper Manufacturing Station TMS-01-0400 (3SAE) (NorthLab Photonics, Sweden), which allows manufacturing tapered fibers with arbitrary shapes, low losses, and excellent repeatability, as has been previously reported^14^. The first inscription process was carried out from a 100 μm—waist tapered fiber. The transitions between the original optical fiber and the uniform waist of the taper were of around 5 mm each, for a total taper length of about 30 mm, as can be seen in Fig. 2.

### Micro-drilled tapered optical fiber (MDTF): 50 µM-waist tapered fiber

Finally, for the second MDTF, a 50 μm-waist was employed. A schematic illustration of this micro-drilled 50 μm–waist tapered optical fiber is depicted in Fig. 2. In this case, the transitions between the original SMF and the uniform 50 μm-waist were also of 5 mm each and a total taper length of 30 mm was also carried out.

## Experimental setup

**Figure 3:**
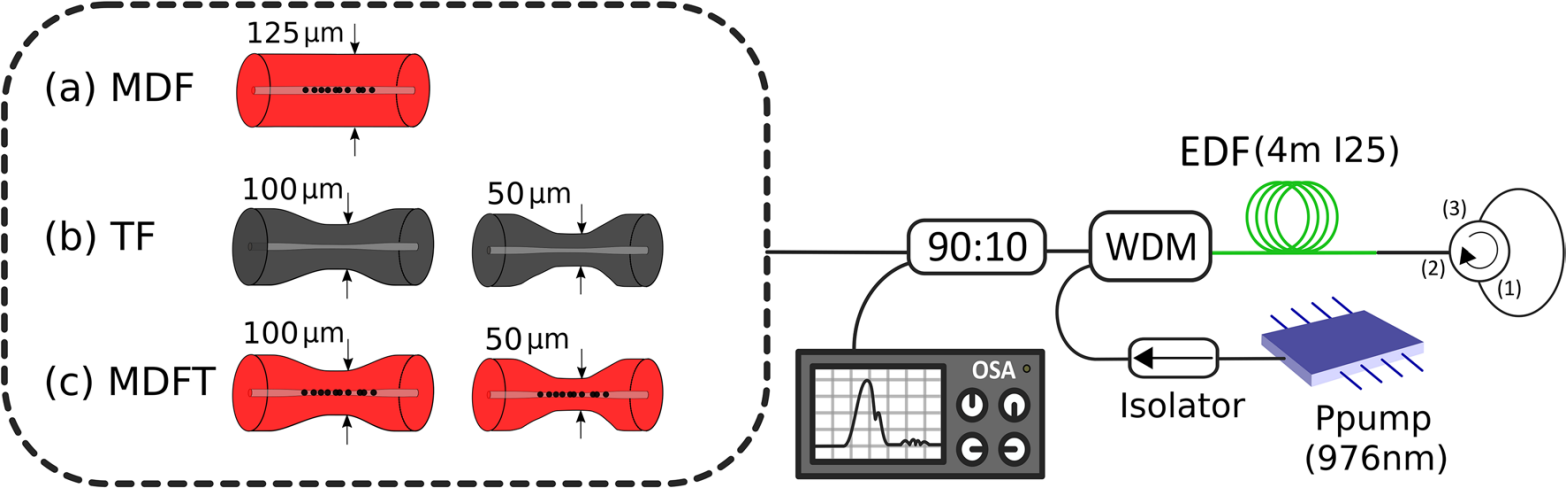
Schematic diagram of the experimental linear short-cavity fiber laser setup, in which (**a**) a micro-drilled optical fiber (MDF), (**b**) un-drilled tapered optical fibers (TF) and (**c**) micro-drilled tapered fibers (MDFT) were used to reflect an amplified signal.

Figure 3 shows a schematic diagram of the experimental setup used to evaluate the laser generation and sensor properties when using the distributed reflectors within a short-linear-cavity fiber acting as a mirror. In this figure, the three types of reflectors employed can be seen: (a) micro-drilled optical fiber (MDF), (b) un-drilled tapered optical fibers (TF), and (c) micro-drilled tapered fibers (MDTF).

A 980/1550 nm wavelength-division multiplexer (WDM) injects the pump laser centered at 976 nm into the linear-cavity fiber laser. The gain medium, located at the common port of the WDM, consists of 4 m of erbium-doped fiber (EDF)^9^. The EDF was the I25 (980/125) (Fibercore Inc.), suitable for C amplifiers with a core composition optimized for erbium-doped fiber amplifiers (EDFAs) in dense wavelength-division multiplexing (DWDM) networks. The peak core absorption ranges from 7.7 to 9.4 dB/m at 1531 nm. The linear cavity of the laser ends at a broadband reflector that consists of a fiber loop mirror (FLM) formed by an optical circulator in which ports 3 and 1 are connected^9^. After this, the recirculating signal travels through the 1550-nm port of the WDM to an optical coupler. At the optical coupler (OC), 10% of the signal was monitored by an optical spectrum analyzer (OSA) with a resolution of 0.03 nm, and the other 90% was guided to the distributed reflectors. All the experimental measurements were carried out at room temperature, and no vibration isolation or temperature compensation techniques were employed.

## Results and discussion

### Laser generation properties

It has been previously demonstrated by the authors that micro-drilling techniques have an enhancing effect not only on the laser generation^9^ but also on the sensor properties^15^. This is evidenced by the results presented in^15^, where some of the lasing and sensor properties when using a single-mode micro-drilled optical fiber (MDF) as a reflector were experimentally demonstrated.

**Figure 4:**
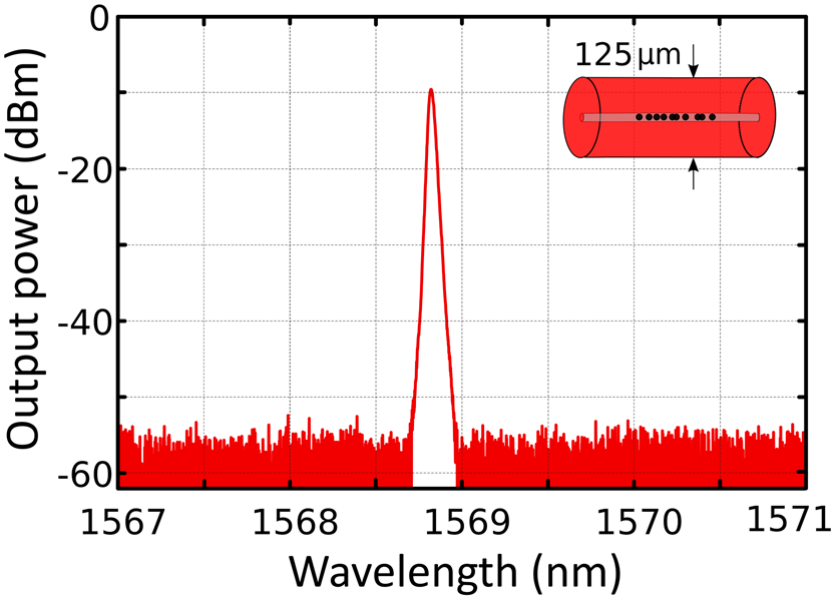
Output spectra of a short-linear-cavity fiber laser when using a single mode MDF pumped by a 976-nm laser at 140 mW.

Figure 4 shows the output spectra of a short-linear-cavity fiber laser using a single-mode MDF as a reflector. In that case, a single-wavelength laser centered at 1568.6 nm with an optical signal to noise (OSNR) level of 45 dB and an output power level of −9.6 dBm were obtained when pumped at 140 mW. In addition to this, this linear short-cavity fiber laser presented an efficiency of about 0.08%. Previous studies demonstrate that these values are reasonably good for utmost sensor applications^16^.

Then, the laser generation properties when using optical tapered fibers as reflectors, both un-drilled (TF) and micro-drilled (MDTF) are presented. Previous experimental studies presented the analysis of the backscattering generation in a 50 μm–waist diameter micro-drilled tapered fiber within a similar linear-cavity fiber laser^9^. In this case, the aim is to analyze in depth the impact of a 100 μm-waist and to evaluate the effect of using different pump power wavelengths.

**Figure 5:**
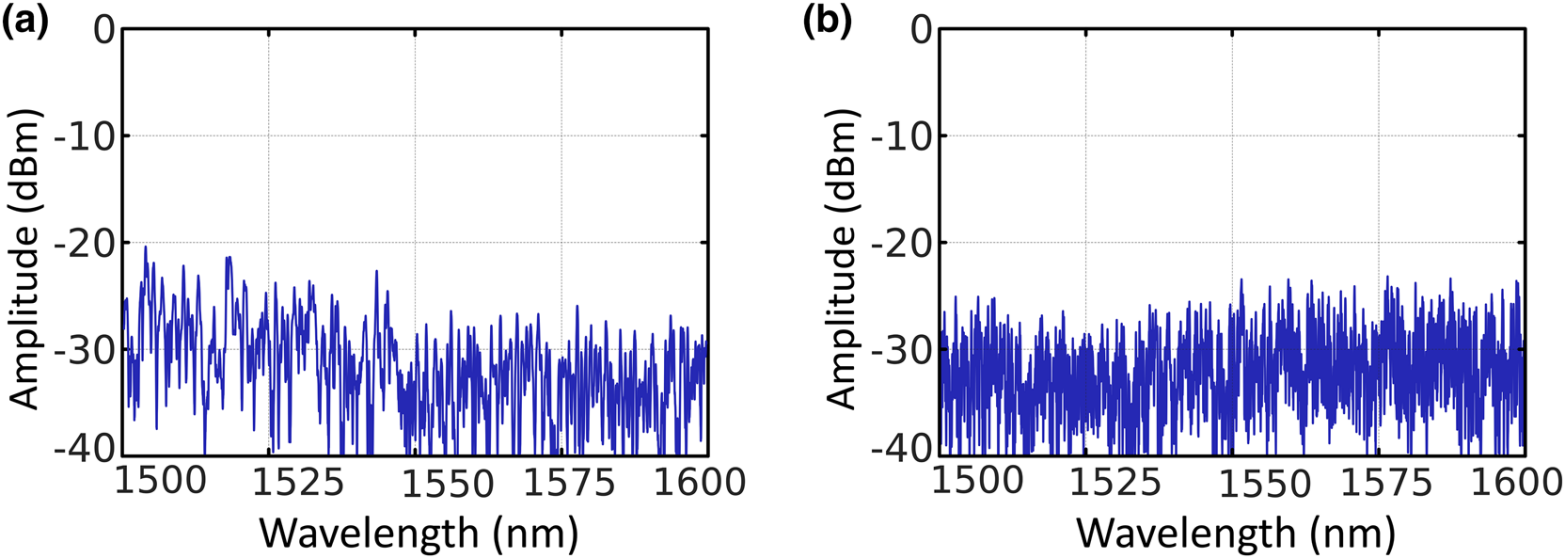
(**a**) Reflection spectrum of the 50 µm-waist micro-drilled tapered fiber. (**b**) Reflection spectrum of the 100 µm-waist micro-drilled tapered fiber.

Figures 5a,b present the reflection spectra of the 50 μm–waist and 100 μm–waist diameter micro-drilled tapered fiber, in that order. These reflection spectra show a fairly flat response, so the emission wavelength of the generated lasers will be determined mainly by the spectrum of the generated amplified spontaneous emission (ASE). For both cases, when the pumping wavelengths are centered at 1445 nm and 1480 nm, the maximum output power level is around 1562 nm.

**Figure 6:**
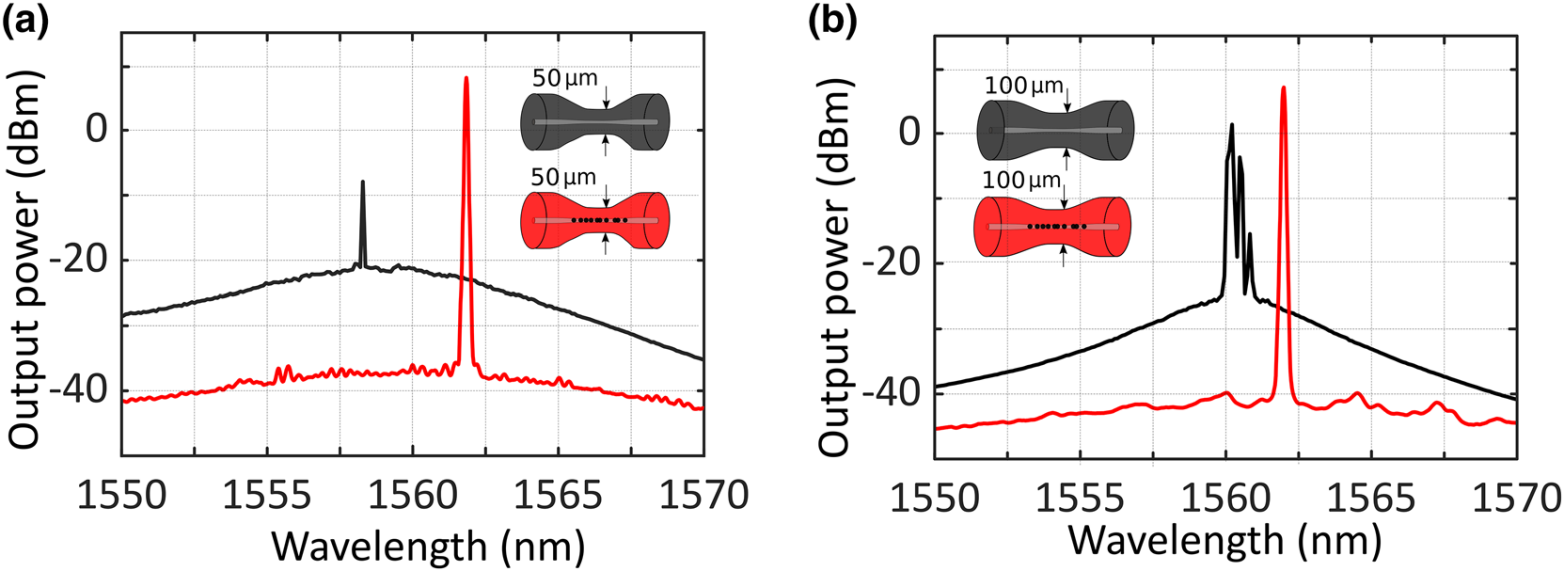
(**a**) Output spectra of the short-linear-cavity fiber laser, pumped by a 976-nm light at 350mW, when using a 50 µm-waist un-drilled (black line) or micro-drilled tapered fiber (red line). (**b**) Output spectra of the short-linear-cavity fiber laser, pumped by a 976-nm light at 350mW when using a 100 µm-waist un-drilled (black line) or micro-drilled tapered fiber (red line).

Figure 6 shows a comparison between the results measured when using a 50 µm-waist or 100 µm-waist tapered fiber, as presented in Fig. 6a,b in that order. Each of these figures shows the output spectra for an un-drilled tapered fiber (black line) or a micro-drilled tapered fiber (red line), both of them being pumped with 350 mW at 976-nm. As it was previously pointed out, the micro-drilling, even with a higher waist diameter and using a different pump laser, dramatically enhances the laser generation properties, as expected, due to the increase of the total reflectivity.

As Fig. 6a shows, when a 50 µm-waist un-drilled tapered fiber was used as reflector (black line), the small-signal gain was not sufficient for single-wavelength lasing. However, when this reflector is replaced by a micro-drilled tapered one, a single-wavelength laser centered at 1561.8 nm was emitted. The output power level obtained from this single-laser oscillation when pumped at 350 mW was 8.2 dBm, with an OSNR of 45 dB (red line). The efficiency of this linear short-cavity fiber laser was about 1.9%.

This experimental comparison was also carried out by using 100 µm-waist tapers, as illustrated in Fig. 6b. In this case, when an un-drilled tapered was evaluated, the obtained output spectrum showed that, one more time, the small-signal gain was not sufficient for single-wavelength lasing and longitudinal-mode competition was observed (black line). On the other hand, when a 100 µm-waist micro drilled taper was used, a single-wavelength laser centered at 1562 nm, with an output power level of 7.2 dBm and an OSNR of 48 dB was measured (red line). In this case, the efficiency, evaluated under the same circumstances, was 1.5%.

**Figure 7:**
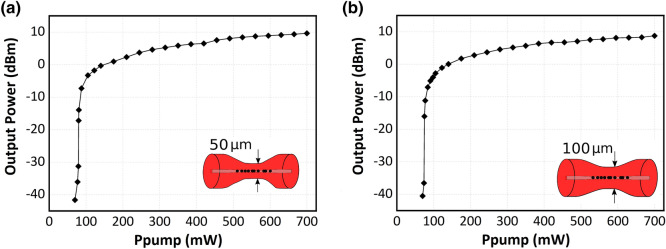
(**a**) Relationship between the output-power values as a function of 976-nm pump power for the 50 µm-waist MDTF-based fiber laser. (**b**) Relationship between the output-power values as a function of 976-nm pump power for the 100 µm-waist MDTF-based fiber laser.

Figure 7a,b display the experimental results of the measured output power level as a function of the inserted 976-nm pump power for the 50 µm-waist and 100 µm-waist tapered fibers respectively, both presenting a similar pump power threshold of about 80 mW.

As it is well known, erbium-doped fibers can be pumped not only by using wavelengths at the 980 nm-band but also around 1480 nm. To further study the properties of this short-linear-cavity laser when pumping at other central wavelengths, the pump laser centered at 976 nm was replaced by others centered at 1445 nm and 1480 nm. Then, a similar experimental study was carried out for these four reflectors.

**Figure 8:**
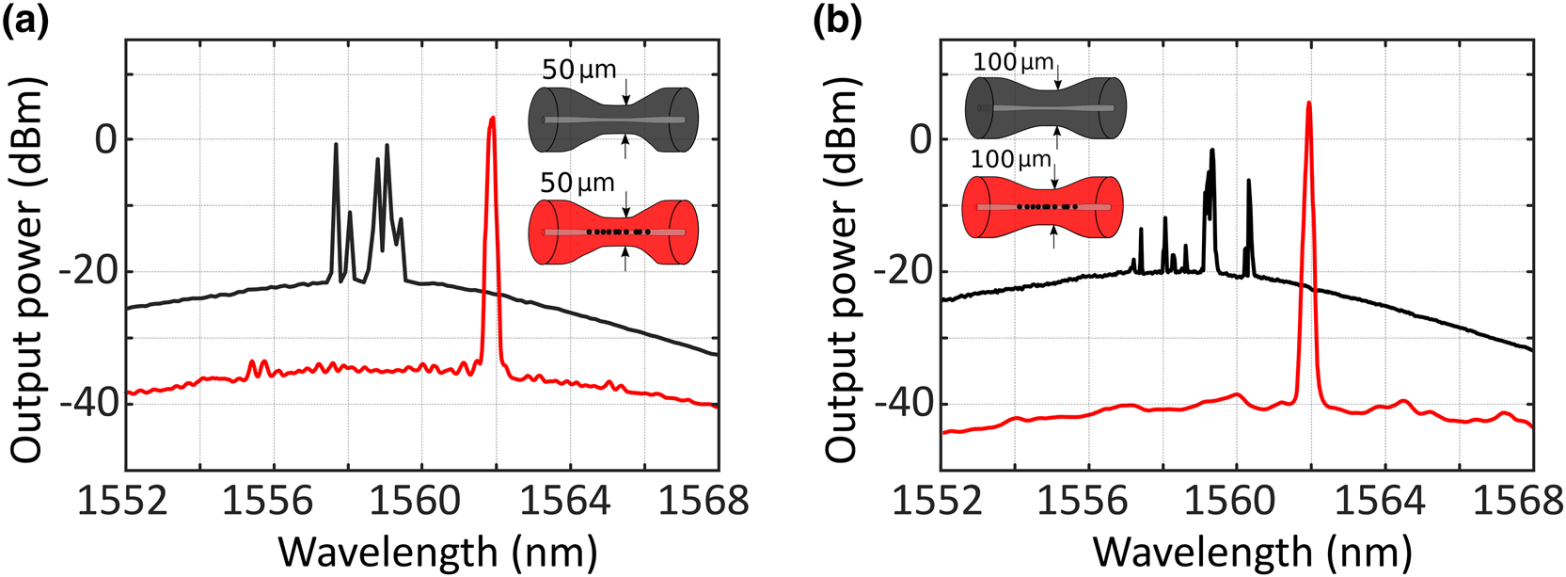
(**a**) Output spectra of the short-linear-cavity fiber laser, pumped by a 1445-nm light at 500 mW and using an un-drilled (black line) or a micro-drilled (red line) 50 µm-waist tapered fiber. (**b**) Output spectra of the short-linear-cavity fiber laser, pumped by a 1445-nm light at 500 mW and using an un-drilled (black line) or a micro-drilled (red line) 100 µm-waist tapered fiber.

Figure 8 shows a comparison between the results attained using a 50 µm-waist or 100 µm-waist tapered fiber, as illustrated in Fig. 8a,b respectively, both of them being pumped with 500mW at 1445-nm light. Once more, the micro-drilling, even with a higher waist diameter and using a different pump laser, dramatically enhances the laser generation properties.

When using an un-drilled reflector, both output spectra of 50 µm and 100 µm waist tapered fibers showed that a small-signal gain, not enough for single-wavelength lasing and longitudinal-mode competition, was observed (see black lines in Figs. 8). Nevertheless, when micro-drilled tapered fibers were used, a single laser emission line can be observed. In the case of using a 50 µm-waist tapered fiber, this single laser emission line was centered at 1561.9 nm, with an output power level of 3.4 dBm and an OSNR of 39 dB. On the other hand, when a 100 µm-waist tapered fiber was used as reflector, this single laser emission line was also centered at 1561.9 nm. However, an output power level of 5.6 dBm and an OSNR of 45 dB were measured.

**Figure 9:**
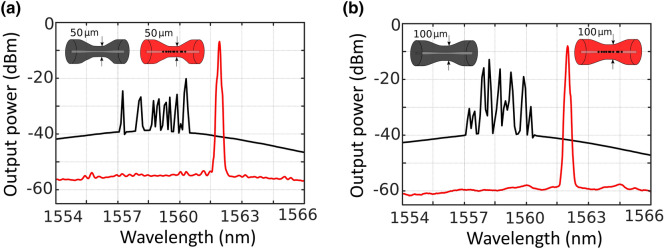
(**a**) Output spectra of the short-linear-cavity fiber laser, pumped by a 1480-nm light at 500 mW and using an un-drilled (black line) or a micro-drilled (red line) 50 µm-waist tapered fiber. (**b**) Output spectra of the short-linear-cavity fiber laser, pumped by a 1480-nm light at 500 mW and using an un-drilled (black line) or a micro-drilled (red line) 100 µm-waist tapered fiber.

The output spectra of the short-linear-cavity fiber laser, when pumped by a 1480-nm light at 500 mW is shown in Fig. 9. As expected, and despite using a different pumping wavelength, the micro-drilling significantly enhances the laser generation properties, as seen in the red lines in both Fig. 9a,b.

When a 50 µm-waist tapered fiber was used as a reflector, a single-wavelength laser centered at 1562 nm, with an output power of −6.9 dBm and an OSNR of 48 dB (Fig. 9a red line). In this case, the optical efficiency of this linear short-cavity fiber laser was as low as 0.04%. On the other hand, a single-wavelength laser also centered at 1562 nm, with an output power of −8 dBm, an OSNR of 51 dB and an optical efficiency of 0.03% were measured when a 100 µm-waist tapered fiber was used as a reflector (Fig. 9b red line). It should be noted that, in this case, although the output power level has been reduced, the noise level has been further reduced, so the OSNR level is increased.

**Figure 10:**
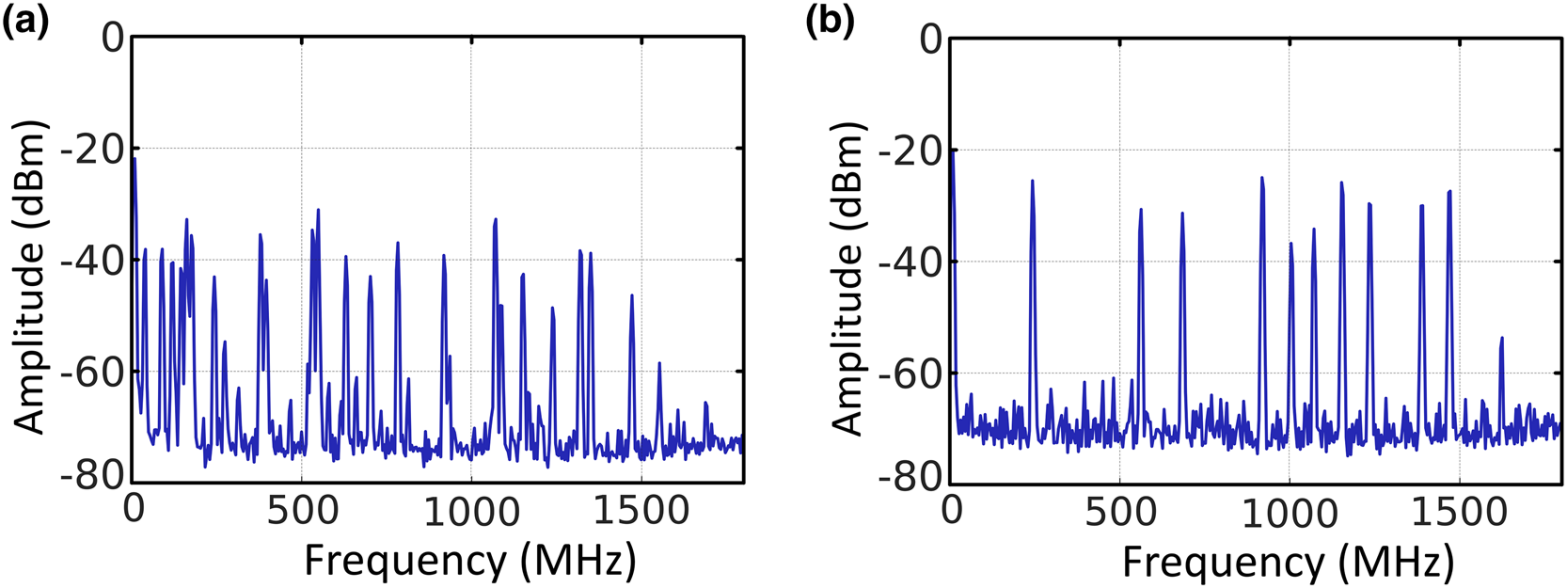
(**a**) Radio-frequency spectra of the beat of the 50 µm-waist tapered fiber with the TLS when pumped at 1480-nm light. (**b**) Radio-frequency spectra of the beat of the 100 µm-waist tapered fiber with the TLS when pumped at 1480-nm light.

In order to experimentally characterize the longitudinal-mode behavior of these lasers, the output port, previously monitored by an OSA, was connected to a photodetector in combination with an electrical spectrum analyzer (ESA) to perform measurements in the electrical frequency domain. The reflected signal from the MDTFs were mixed with the signal form a tunable laser source (TLS, Agilent 8164B), though a 3 dB coupler to perform a heterodyne detection. Figures 10a,b illustrate the frequency spectra corresponding to the frequency domain conversion, when using a 50 µm-waist and 100 µm-waist tapered fiber, respectively, both pumped by a 1480-nm light. These two figures clearly show the appearance of multiple longitudinal mode beating so, presenting a multimode operation. Similar results were obtained when pumped by 976-nm and 1445-nm light.

It has been experimentally demonstrated that the obtained output spectra when using an un-drilled tapered fiber (TF) presents a small-signal gain because of its low reflectivity. However, it is not sufficient enough for single-wavelength lasing and, in addition to this, longitudinal mode competition was observed for all the different pumping wavelengths used in this work. For this reason, this TF reflector will not be analyzed in the following studies.

Table 1 contains, in summary form, a comparison of the results obtained in the experiments. There, it is presented a comparison of the laser generation properties using a micro-drilled fiber, a 50 or a 100 µm-waist micro-drilled tapered fiber as a reflector, as a function of the inserted pump power wavelength.

**Table 1:** Comparison of laser generation properties when using a micro-drilled fiber (MDF), a 50 μm-waist or a 100 μm-waist micro-drilled tapered as a reflector (MDTF).

Optical fiber structure reflector	Pump power wavelength	Central wavelength	Output power	OSNR	Efficiency (%)
MDF	976 nm	1568.6 nm	−9.6 dBm	45 dB	0.08
50 μm-waist MDTF	976 nm	1561.8 nm	+ 8.2 dBm	45 dB	1.89
	1445 nm	1562 nm	+ 3.4 dBm	38 dB	0.44
	1480 nm	1562 nm	−6.9 dBm	48 dB	0.04
100 μm-waist MDTF	976 nm	1562 nm	+ 7.2 dBm	48 dB	1.5
	1445 nm	1561.9 nm	+ 5.8 dBm	45 dB	0.76
	1480 nm	1562 nm	−8 dBm	51 dB	0.03

### Temperature and strain sensor

Considering the above results, both in terms of output power, OSNR level as well as optical efficiency, the properties of these reflectors as temperature and strain sensors were also analyzed. The following studies were carried out using a micro-drilled optical fiber (MDF), a 50 µm-waist MDTF and a 100 µm-waist MDTF all of them with a pump power wavelength of 976 nm.

First, this structure was characterized as temperature sensor when no strain was applied. The wavelength-shift sensitivity to temperature was characterized using the MDF, the 50 µm-waist MDTF and the 100 µm-waist MDTF distributed reflectors. This characterization was carried out by using a climatic chamber in the range of 35 $^\circ$C to 100 $^\circ$C and taking samples each 2 $^\circ$C.

**Figure 11:**
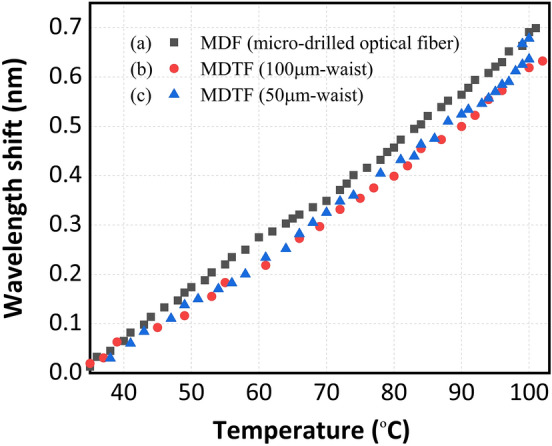
Wavelength shift as function of temperature when using (**a**) the MDF, (**b**) the MDTFs with 100 μm-waist or (**c**) the 50 μm-waist as temperature sensor.

As depicted in Fig. 11, the center wavelength-shift for these three single-wavelength lasers when using a MDF, a MDTF with 100 μm-waist or a MDTF with 50 μm-waist, (see Fig. 11a,b,c, respectively) present a clear linear behavior (the mean square errors were equal to 0.9983, 0.9953 and 0.9976 respectively) and temperature sensitivities of 10 pm/$^\circ$C, 9.1 pm/$^\circ$C and 9.6 pm/$^\circ$C were measured, in that order. The attained values were close to the typical value for temperature-induced Bragg wavelength shift in silica fibers operating at 1550 nm, which is around 11 pm/$^\circ$C^5^.

In the view of the above, these sensor heads based on micro-drilled optical fibers, were placed in a high precision single-axis motorized stage (MS) in order to evaluate the wavelength shift induced by axial-strain changes. The characterization of the sensor heads consisted of 29 steps of 3.15 µε per step.

As it is well known strain, unlike temperature, is a process mainly driven by the mechanical deformation of the optical fiber when a longitudinal stretch is applied. The taper sensitivity to strain is mainly determined by the physical dimensions of the sensor system, as it has been previously studied and demonstrated^17^. This deformation is inversely proportional to the radius of the optical fiber, so the smaller the diameter, the greater the effect of the strain applied on the sensing part. The strain loads applied to a FBG and a tapered fiber are related according to:

$$\Delta {\varepsilon }_{FBG} E {A}_{FBG}= \Delta {\varepsilon }_{taper} E {A}_{taper}$$

where ${\varvec{E}}$ is the Young modulus of the sensor material, ${A}_{FBG}$ and ${A}_{taper}$ are the cross-sectional areas in the FBG and in the tapered optical fiber, respectively, and ${\varepsilon }_{FBG}$ and ${\varepsilon }_{taper}$ are the strain applied on the FBG or the tapered fiber, in that order^17^. Due to the refractive index modifications caused by the inscription process, the mechanical and optical properties of the material in a FBG or the tapered optical fibers are not the same. So, the strain applied to these reflectors will depend not only on the ratio of the cladding diameters but also to the mechanical properties of each one of them. Consequently, in a complex structure such as these micro-drilled tapered fibers (MDTFs), not only the dimensions of the tapered fiber waist affect, but also the transitions between the original optical fiber and the uniform waist of the taper or the refractive index modifications among others, also modify the response of the sensor.

In order to quantify the effect only of those refractive index modifications and not the fiber core dimensions, the first experiment carried out was with a micro-drilled standard single-mode optical fiber, with a diameter of 125 microns, in order to compare its response with that of a FBG, also with the same diameter, and to see if these inscriptions provided any improvement in terms of the sensor's sensitivity. After analyzing this first structure with a size similar to that of a FBG, a more detailed study was carried out for reflectors with smaller waist diameters, from 125 µm to 100 µm and 50 µm.

**Figure 12:**
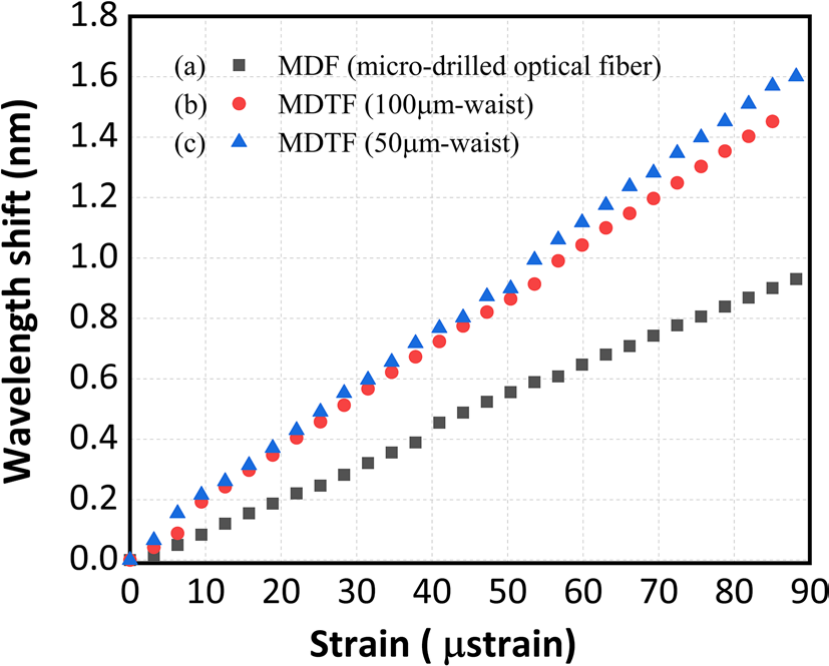
Wavelength shift as a function of strain change when using (**a**) a MDF, (**b**) a MDTF with 100 μm-waist, and (**c**) a MDTF with 50 μm-waist.

Figure 12 presents the central emission wavelength shift when the structure was subjected to the abovementioned strain variations using (a) a MDF, (b) a MDTF with 100 μm-waist, and (c) a MDTF with 50 μm-waist. These results show linear response as evidenced by the R-squared values, close to 1 (0.9983, 0.9991 and 0.9992 respectively), showing sensitivities as good as 10.9 pm/µε, 17 pm/µε and 18.1 pm/µε for the MDF, the MDTFs with 100 μm-waist or 50 μm-waist, in that order.

These values, when compared with the typical value for strain-induced Bragg wavelength shift that is approximately 1.2 pm/µε^5,6^, present a significant enhancement of more than one order of magnitude. This represents a substantial improvement of the strain sensitivity.

Finally, Table 2 provides a summary of the data acquired over this experimental study. This table shows a temperature and strain comparison for the sensor heads of interest as well as the typical values for temperature and strain induced Bragg sensor. Although temperature sensitivities achieved were similar to FBGs, the strain sensitivity presents more than one order of magnitude enhancement.

**Table 2:** Comparison of temperature and strain sensitivity for a FBG, micro-drilled fiber or micro-drilled tapered fiber.

Optical fiber structure	Waist diameter	Temperature sensitivity	Strain sensitivity
FBG^5^	125 µm	11 pm/$^\circ$C	1.2 pm/µstrain
MDF	125 µm	10.5 pm/$^\circ$C	10.9 pm/µstrain
MDTF	100 µm	9 pm/$^\circ$C	17 pm/µstrain
MDTF	50 µm	9.5 pm/$^\circ$C	18.1 pm/µstrain

## Conclusions

In conclusion, this work presents an experimental performance analysis of different types of quasi-randomly distributed reflectors written into single-mode fiber, when used as sensing mirrors or sensor heads in a short-linear-cavity fiber laser. In particular, the features of the laser generation when optical tapered fibers (TF), micro-drilled optical fibers (MDF) or two different micro-drilled tapered fibers (MDTF) are presented. Regarding the laser generation properties, the best results in terms of output power level, OSNR, or optical efficiency, were obtained for the case of micro-drilled tapered optical fiber when pumped at 976 nm. In particular, when the 50 μm-waist or 100 μm-waist micro-drilled tapered optical fibers were used, a single-wavelength reflector laser centered at 1561.8 nm or 1562 nm, with an output power level of 8.2 dB or 7.2 dB and an OSNR higher than 45 dB or 48 dB were measured respectively.

In terms of the properties as a sensor, temperature sensitivities of around 10 pm/$^\circ$C were measured. However, and even though the achieved temperature sensitivities were close to the typical value for temperature-induced Bragg wavelength shift in silica fibers operating at 1550 nm, the strain sensitivity was significantly improved. As an example, when the 50 µm-waist MDTF was employed as distributed reflector a strain sensitivity as high as 18.1 pm/µε was measured, which means an improvement of more than one order of magnitude in comparison to FBG sensors.
